# In Silico Screening Identification of Fatty Acids and Fatty Acid Derivatives with Antiseizure Activity: In Vitro and In Vivo Validation

**DOI:** 10.3390/pharmaceutics16080996

**Published:** 2024-07-27

**Authors:** Emilia Mercedes Barrionuevo, Estefanía Peralta, Agustín Manzur De Nardi, Juliana Monat, Maximiliano José Fallico, Manuel Augusto Llanos, Luciana Gavernet, Emilio Román Mustafá, Pedro Martin, Alan Talevi

**Affiliations:** 1 Laboratory of Bioactive Compound Research and Development (LIDeB), Faculty of Exact Sciences, National University of La Plata (UNLP), Blvd. 120 1489, La Plata 1900, Argentina; 2 Argentinean National Council of Scientific and Technical Research (CONICET), CCT La Plata, La Plata 1900, Argentina; 3 Instituto de Estudios Inmunológicos y Fisiopatológicos (IIFP), Universidad Nacional de La Plata–CICPBA–CONICET, Boulevard 120 no. 1489, La Plata 1900, Argentina; 4 Electrophysiology Laboratory of the Multidisciplinary Institute of Cell Biology [Argentine Research Council (CONICET), Scientific Research Commission of the Province of Buenos Aires (CIC-PBA) and National University of La Plata (UNLP)], La Plata 1900, Argentina

**Keywords:** epilepsy, fatty acids, free fatty acids, molecular docking, molecular dynamics, NaV1.2, ketogenic diet, nutraceuticals, CaV2.2, CaV3.1

## Abstract

High fat diets have been used as complementary treatments for seizure disorders for more than a century. Moreover, many fatty acids and derivatives, including the broad-spectrum antiseizure medication valproic acid, have been explored and used as pharmacological agents to treat epilepsy. In this work, we have explored the anticonvulsant potential of a large library of fatty acids and fatty acid derivatives, the LIPID MAPS Structure Database, using structure-based virtual screening to assess their ability to block the voltage-gated sodium channel 1.2 (NaV1.2), a validated target for antiseizure medications. Four of the resulting in silico hits were submitted for experimental confirmation using in vitro patch clamp experiments, and their protective role was evaluated in an acute mice seizure model, the Maximal Electroshock seizure model. These four compounds were found to protect mice against seizures. Two of them exhibited blocking effects on NaV1.2, CaV2.2, and CaV3.1.

## 1. Introduction

Epilepsy is one of the most prevalent chronic disorders of the central nervous system, ranking among the first 25 causes of Disability Adjusted Life Years (DALYs) between 0 and 24 years of age [1]. Although there are currently around 30 antiseizure medications available on the market [2,3,4], roughly 30% of patients are unable to control epileptic seizures based on pharmacological intervention alone [5,6], and these numbers have remained approximately constant for more than a century [7]. Patients that fail to become seizure-free with medications may be candidates for resective surgery or resort to adjunctive complementary treatments, such as the ketogenic diet, vagus nerve stimulation, or behavioral interventions, which may improve life quality and seizure control [8,9].

The anticonvulsant effects of various fatty acids [10,11,12,13] (including the broad spectrum approved antiseizure agent valproic acid), and fatty acid derivatives [14,15] have been reported. These phenotypic effects could be attributed to various modes of action associated with antiseizure effects, including the inhibition of voltage-dependent sodium and calcium currents [16,17] and GABAA receptors [15,17], among other molecular mechanisms. Furthermore, fatty acids have a proven modulatory activity on inflammatory processes, through very diverse mechanisms that involve the interaction with membrane receptors, the action on intracellular signaling pathways, transcription factors, and gene regulation, and the impact of the lipid composition of the cell membrane on the amount and type of eicosanoids that are produced (and therefore on the pro- and anti-inflammatory mediators that are generated) [18,19,20,21].

The role of fatty acids as modulators of inflammation and their antiseizure effects are of great interest for two reasons. First, it is well established that neuroinflammation (and perhaps even systemic inflammation) plays a key role in epileptogenesis [22,23,24] and epilepsy [25]. In fact, epilepsy has sometimes been described as a vicious cycle in which seizures produce inflammation that, in turn, could facilitate the occurrence and severity of new seizures [26]. Therefore, a therapy that had antiseizure and anti-inflammatory effects at the same time could interrupt the vicious circle in either of these two instances, perhaps contributing to a better progression of the disease. On the other hand, the ketogenic diet, based on a high fat and low carbohydrate and protein diet, has been used as a complementary therapy for epilepsy for more than a century [27], while other diets based on polyunsaturated fatty acids have been evaluated against epilepsy with mixed results [28]. It is possible that part of the therapeutic success of such diets is explained by the intrinsic activity of the fatty acids included in the lipid content of the diet, and that diets with improved efficacy can be designed by enrichment with fatty acids with antiseizure and anti-inflammatory activity. Therefore, active fatty acids and their derivatives have potential both from a pharmacological point of view and from a nutraceutical perspective.

Here, and based on the previous reports on the inhibitory effects of fatty acids against voltage-operated sodium channels, we have implemented a structure-based virtual screening campaign on the LIPID MAPS Structure Database [29]. Four candidates were later submitted to an in vivo acute model of seizure and in vitro assays to confirm the in silico predicted affinity for the molecular target.

## 2. Materials and Methods

### 2.1. Reactants

2-amino-octanoic acid, 5-hexenoic acid, 9-hydroxydecanoic acid, and 9-phenylnonanoic acid (PH009756), CsF, CsCl, HEPES, EDTA, MgATP, CaCl_2_, BaCl_2_, tetraethylammonium chloride (TEA), chloride, and EGTA were acquired from Sigma Aldrich (Steinheim, Germany). Phenytoin was acquired from Fluka Analytical (Buchs, Switzerland). Geneticin was purchased from Santa Cruz biotechnology (Dallas, TX, USA). All other reagents were of analytical grade and were purchased from local suppliers.

### 2.2. Structure-Based Virtual Screening

A previously reported optimized model of the human NaV1.2 (hNaV1.2) channel, based on the experimental structure of this target (PDB-ID: 6J8/E) was used for the structure-based in silico screening [30]. The receptor was prepared for molecular docking using the UCSF Chimera 1.15 software [31].

A database of 2858 fatty acid structures and derivatives was downloaded from the LIPID MAPS Structure Database (LMSD, 2021-08-03) [29]. The ligands were prepared for molecular docking using OpenBabel 3.1.1 [32] and AutoDock Tools 1.5.6 [33]. The protonation state was corrected to a pH of 7.4, and a previous conformational optimization was performed using the steepest descent algorithm and 2500 minimization steps. The docking site was defined with a 22 × 22 × 35 Å box that included the entire pore. Docking was performed using AutoDock Vina 1.2.0 [34,35], with an exhaustivity of 64 and 9 poses generated per ligand.

A retrospective screening experiment was employed to evaluate the performance of the docking protocol. A mixed validation set composed of active and inactive compounds was compiled. Active molecules were selected from isoform-specific patch clamp experiments reported in the literature [36,37,38,39,40,41,42,43,44,45,46,47,48,49,50,51,52,53] with IC50 values lower than 10 µM. Inactive compounds were selected from ChEMBL26, considering compounds with an IC50 value greater than 10 µM in the batrachotoxin (BTX) displacement assay. The validation sets consisted of 98 active and 700 inactive compounds (Table S1). The area under the receiver operating characteristic curve (AUC-ROC) metric was calculated using LIDeB tools’s Metrics [54] (results shown in the Supplementary Materials, Figure S1).

The docking poses of those compounds with the greatest ligand efficiency were visually inspected to assess specific interactions within the pore. After considering previous toxicity and activity reports, along with chemical and physical properties, and availability, four compounds were prioritized for experimental testing: 5-hexenoic acid, an unsaturated fatty acid, and 2-aminooctanoic acid, 9-phenylnonanoic acid, and 9-hydroxydecanoic acid, three fatty acid derivatives.

9-phenylnonanoic acid and 9-hydroxydecanoic acid were also subjected to molecular dynamics (MD) simulations to explore the interactions with NaV1.2 by sampling the conformational space around the initial docking-generated complexes. Both protein−ligand complexes were embedded in a POPC membrane patch using the CHARMM-GUI server [55,56], solvated in a rectangular box of TIP3P water molecules, and sufficient NaCl was added to mimic 0.15 mM salt concentration. Simulations were run with the OpenMM simulation package, using the ff19SB force field for the protein, the Lipid21 force field for membrane lipids, and OpenFF for ligands. First, each complex was energy minimized by running 2500 steps of steepest descent followed by 2500 steps of conjugated gradient. Then, equilibration and heating processes were run in six consecutive steps, starting with an NVT ensemble and then switching to an NPT ensemble while progressively releasing the constraints over the protein and lipids. Simulations were run at 303.15 K, using Langevin dynamics with a weak coupling as a thermostat. Pressure control was achieved using a Berendsen barostat with semi-isotropic coupling. Long-range electrostatics were calculated using the PME algorithm with a cutoff of 10 Å. For production runs, a time-step of 4 fs was achieved by combining the hydrogen mass repartitioning and the SHAKE algorithms [57,58]. Three independent simulations of 25 ns were run for each complex, in order to improve the conformational sampling of the MD.

### 2.3. In Vivo Assays

The selected in silico hits were evaluated using the murine Maximal Electroshock seizure (MES) test to assess their antiseizure activity [59]. Adult male specific-pathogen-free BALB/c mice provided by the Faculty of Veterinary Sciences, National University of La Plata (FCV-UNLP), were used. Animals were housed in cages with 4 animals per cage, under controlled temperature and a 12 h light/dark cycle, provided with food and water ad libitum, and environmental enrichment. A minimum period of six days was given after transport for physiological acclimatization before any procedure. Over the five days before the assay, mice were subject to procedural acclimatization by administering 0.1 mL of saline daily, i.p. All the tests were conducted during the light cycle.

Four groups of mice (N = five per group) were used per test compound. Each test compound was administered i.p. and tested at two doses (30 and 100 mg/kg) at 0.5 or 4 h before electrical stimuli, following standard procedures by the US National Institute of Health’s Anticonvulsant Screening Program [60] (the Program has been restructured and renamed as the Epilepsy Therapy Screening Program, which keeps the MES test as one of its primary screening tools).

Fresh solutions were prepared using saline (10 mL/kg) or DMSO (2 mL/kg, BIOPACK) as vehicles. Control mice received vehicles (0.5 h before evaluation) (negative control, N = 5 per vehicle) or the standard anticonvulsant phenytoin (FLUKA, 15 mg/kg, 2 h before evaluation) (positive control, N = 5). The rotarod test was performed to detect any signs of motor impairment associated with neurotoxicity [59], 15 min prior to electrical stimuli. A failed test was considered as the loss of balance on the rotating rod at 6 rpm, during three consecutive tests. Auricular electrodes were placed using a conduction gel to ensure electrical contact, and a 0.2 s pulse of 50 mA, 60 Hz current using a UGO BASILE pulse generator (57800 ECT Unit). In this model, seizures are characterized by a short period of tonic flexion, followed by tonic extension of the hind limbs, typically lasting more than 3 s [61]. The absence of the tonic extension phase is considered as the protection criterion.

After the test, all animals were euthanized using CO_2_. All tests were performed according to protocols approved by the Ethics Committee for Animal Experimentation of the Faculty of Exact Sciences of the University of La Plata, Argentina (FCE-UNLP) (protocol approval number Protocol Number 014-06-15).

### 2.4. Patch Clamp Experiments

Na+ and Ca2+ currents were recorded using the patch clamp technique in the whole-cell and voltage-clamp configuration in HEK cells heterologously expressing the channel of interest using an Axopatch 200 amplifier (Axon Instruments, Molecular Devices, CA, USA). Solutions were perfused via gravity at a flow rate of approximately 1 mL/min. All recordings were performed at room temperature (approximately 24 °C).

For in vitro experiments, 9-hydroxydecanoic acid, 9-phenylnonanoic, and 5-hexenoic acid were dissolved in dimethyl sulfoxide (DMSO), whereas 2-amino-octanoic acid was dissolved in a physiological solution. Fresh aliquots of the fatty acid stock solutions were added to the experimental solutions on the day of the experiment. An appropriate amount of vehicle was added to all control solutions without drugs.

#### 2.4.1. NaV1.2 and NaV1.1 Current Inhibition

Na+ currents were recorded in HEK293 cells stably expressing the human NaV1.1 or the NaV1.2 α-subunit (a kind gift from GlaxoSmithKline, Stevenage, UK), with the same experimental conditions and voltage protocols as previously reported for hNaV1.2 [60]. Cells were cultured in high-glucose minimum essential medium (DMEM), containing 10% fetal bovine serum (Gibco), and 0.5% Geneticin G418 sulfate, in a 5% CO_2_ atmosphere at 37 °C.

Cells were detached using Tryple (Thermofisher) and settled in a glass-bottom experimental chamber with an extracellular bath solution (BS) containing (in mM) NaCl 140, CaCl_2_ 2, MgCl_2_ 1, HEPES 10, and glucose 11; pH was adjusted to 7.4 with NaOH. Glass pipettes were drawn with a resistance ranging from 1.5 to 3.0 MΩ and filled with a solution containing (in mM) CsF 100, CsCl 40, EGTA 10, HEPES 10, NaCl 5, MgCl_2_ 2, Na_2_ATP 4; the pH was adjusted to 7.3 with CsOH. Currents were filtered with a 4-pole low-pass Bessel filter at 2 kHz and digitized at a sampling frequency of 200 kHz (Digidata 1440, Molecular Devices, CA, USA).

The Na+ current amplitude and series resistance stability were evaluated with a test pulse protocol from −80 mV to −10 mV for 15 ms and repeated every 5 s. Once stability was achieved (approx. 10 min), the steady-state inactivation voltage protocol (see below) was applied under control conditions (vehicle) and in the presence of each fatty acid at a 100 µM concentration. This protocol consists of a double voltage step sequence where a series of pre-conditioning steps (from −130 to −40 mV) are applied for 2.5 s, each followed by a test pulse of 25 ms to −10 mV. The magnitude of the current evoked by the test pulse depends on the available fraction of channels to be opened after each pre-conditioning pulse. The available fraction of channels at each pre-conditioning potential was calculated as the relationship between the peak current measured in the test pulse preceded by each pre-conditioning step (I) and that evoked after a pulse of −130 mV in which the current, and the available fraction, was maximal (Imax). Then, the h curves were plotted as the available fraction vs. pre-pulse voltage and fitted using the following Boltzmann equation:

(1) 1Imax=11+eV12−Vk,

where *V*_1/2_ is the membrane potential for which the available fraction is 0.5, and *k* is the slope parameter.

The data obtained from the steady-state inactivation voltage protocol were also used to analyze whether the compounds were able to bind to the closed state of the NaV channel, producing a voltage-independent current inhibition. For all the tested fatty acids, the fraction of channels in the closed state (available) was maximal when the pre-conditioning potential was −130 mV. Thus, the Na+ current inhibition observed in the test pulse in this condition allows us to quantify the inhibitory effect on closed or resting channel state.

#### 2.4.2. CaV2.2 and CaV3.1 Current Inhibition

Human Embryonic Kidney (HEK) 293T cells were grown in Dulbecco’s modified Eagle’s medium (DMEM, Gibco) with 10% fetal bovine serum (FBS, Internegocios). Cells were transfected with plasmids containing voltage-gated calcium channel subunit CaV2.2 (Cacna1b, GenBank accession no. AF055477) auxiliary subunits CaVβ3 (Cacnb3, GenBank accession no. M88751) and CaVα2δ1 (Cacna2d1, GenBank accession no. AF286488), or with CaV3.1 (Cacna1g, GenBank accession no. AF190860). To identify transfected cells, an eGFP (enhanced green fluorescent protein)-containing plasmid was used. Transfections were performed using Lipofectamine 2000 (Invitrogen) and Opti-MEM (Gibco). Lipofectamine was used according to the manufacturer’s specifications. We used 0.625 µg of total cDNA and 1.25 µL of lipofectamine per well (growth area 1.9 cm^2^). Transfected cells were cultured for 24 h to allow CaV2.2 expression or for 48 h for CaV3.1 expression. On the day of the experiment, cells were detached from the culture disk with 0.25 mg/mL 1 trypsin (Microvet), rinsed twice, and kept at room temperature (24 °C) in DMEM.

Calcium currents of transiently transfected HEK293T cells were obtained using the internal pipette solution, which contained (in mM) 134 CsCl, 10 EGTA, 1 EDTA, 10 HEPES pH 7.2, and 4 MgATP, with CsOH; the external solution contained (in mM) 2 CaCl_2_ (or 10 BaCl_2_), 1 MgCl_2_, 10 HEPES pH 7.4, and 140 choline chloride (or 140 TEA Chloride), with CsOH. Data were sampled at 20 kHz and filtered at 10 kHz (−3 dB). Recording pipettes with resistances between 2 and 4 MΩ were used and filled with internal solution. Series resistances of less than 6 MΩ were admitted and compensated by 80% with a 10 μs lag time. We discarded cells with a leak current higher than 100 pA at holding potential and leak current was subtracted online using a P/-4 protocol. The test pulse protocol for CaV2.2 recordings consisted in square pulses applied from −100 to +10 mV for 30 ms every 10 s. The test pulse protocol for CaV3.1 recordings consisted in square pulses applied from −100 to −20 mV for 200 ms every 10 s.

### 2.5. Statistics

Data were analyzed and visualized using OriginPro 9 (Origin-Lab), Clampfit (Molecular Devices), and GraphPad Prism 8 (GraphPad Software Inc., San Diego, CA, USA) software version 8.0.1 for Windows. All results are expressed as mean ± standard error of the mean (SEM). A one-sample *t*-test against a hypothetical value of 1 (normalized current value) was applied to assess the statistical significance of the current inhibition, while the same test against a hypothetical value of 0 mV was used to evaluate the statistical significance of ΔV1/2. A paired Student’s *t*-test was applied to compare inhibition between NaV channels isoforms. The activity of the fatty acids on NaV channels was compared using either one-way ANOVA followed by Holm-Sidak’s multiple comparisons test, or the Kruskal–Wallis test, depending on the normality of the data distribution. A *p*-value lower than 0.05 was considered to establish statistically significant differences in all cases.

## 3. Results

### 3.1. Virtual Screening

#### 3.1.1. Validation

Retrospective in silico screening on the compiled database yielded an AUCROC of 0.8017 ± 0.0086, which is a good result considering that an ideal model that could perfectly discriminate activity from inactivity would have an AUCROC value of 1.0, while a model that did not differ in its discriminating power from random selection would have an AUCROC value of 0.5. This result seemed sufficient to proceed to the prospective screening. The ROC curve obtained is included in the supplementary materials (Figure S1).

#### 3.1.2. Molecular Docking

**Figure 1:**
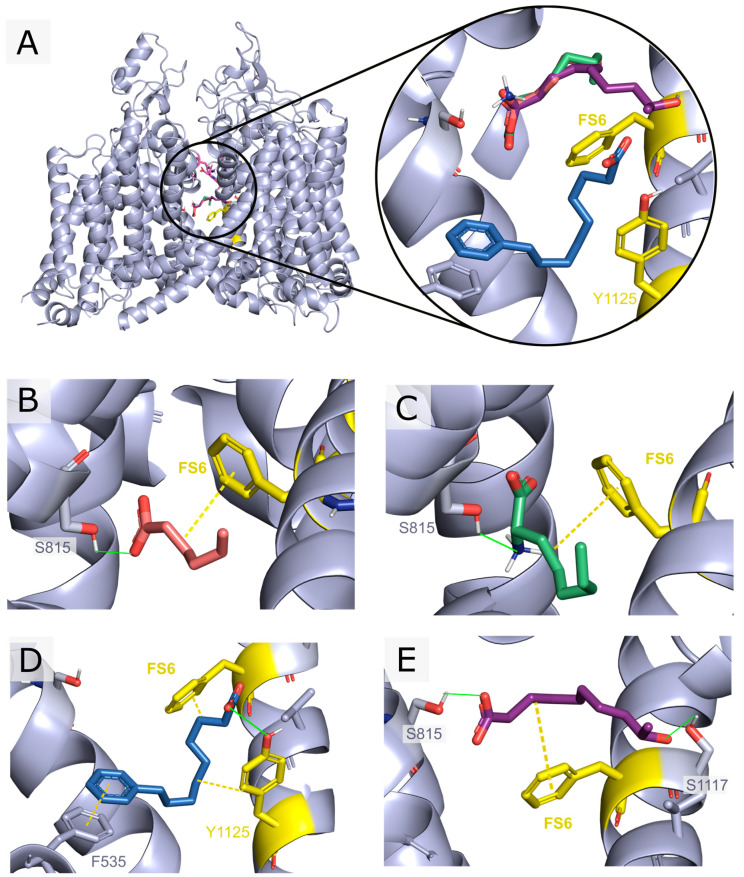
Docking poses vs. hNaV1.2 of the selected in silico hits submitted to in vivo and in vitro assays. The top scoring pose for each ligand is shown. Residues constituting the DEKA ring are represented in pink, while residues F1118 (FS6) and Y1125 from the Ab site are depicted in yellow. Compounds were selected based on the ligand’s efficiency, visual inspection of the poses, and favorable drug-like properties: 5-hexenoic acid (orange), 2-aminooctanoic acid (green), 9-phenylnonanoic acid (blue), and 9-hydroxydecanoic acid (violet). (**A**) Front view of hNaV1.2 along with the top scoring docking poses for the selected compounds. (**B**) 5-hexenoic acid’s highest score docking pose showing a 2.7 Å hydrogen bond with SER815 and a 3.7 Å minimal distance to FS6. (**C**) 2-aminooctanoic acid’s highest score docking pose showing a 1.9 Å hydrogen bond with SER815 and a 3.6 Å minimal distance to FS6. (**D**) 9-phenylnonanoic acid’s highest score docking pose showing a 3.6 Å pi-interaction with PHE535, a 3.0 Å hydrogen bond with TYR1125, and a 4.9 Å minimal distance to FS6. (**E**) 9-hydroxydecanoic acid’s highest score docking pose showing a 2.7 Å hydrogen bond with SER815, a 2.2 Å hydrogen bond to SER1117, and a 3.6 Å minimal distance to FS6.

Docking simulations provided a ranking of the compounds from LMSD, according to their predicted binding affinity to hNaV1.2. Given the variable size of fatty acids and, in particular, the small size of short- to medium-chain fatty acids, compounds were also ranked by their ligand efficiency. Within the receptor pore, the analysis of the docking poses was made with special focus on the small molecule’s high affinity binding site (Ab), which overlaps with the local anesthetic binding site [62]. The latter is characterized by the presence of a conserved phenylalanine residue on helix S6 of domain IV, residue F1118 (FS6), and a conserved tyrosine residue seven positions towards the C-terminal end (residue Y1125). This site is conserved along subtypes NaV1.1-9 of the mammalian channels [63]. The selectivity-filter ring Asp-Glu-Lys-Ala (DEKA ring) found at the outer pore was also highlighted as a reference. Four of the compounds among the top thirty scoring hits (2-amino-octanoic acid, 5-hexenoic acid, 9-hydroxydecanoic acid, and 9-phenylnonanoic acid) (Figure 1) were selected and submitted for in vivo and in vitro characterization.

### 3.2. In Vivo Assays

All tested compounds exhibited antiseizure activity in the MES test. The anticonvulsant profiles are summarized in Table 1. None of the tested compounds showed signs of neurotoxicity on the rotarod test. None of the animals, including those in the control groups, died during the experiments. All mice treated with phenytoin (positive control) were protected from seizures. Contrariwise, seizures were observed in all the animals treated with vehicles (negative controls).

### 3.3. Patch Clamp Experiments

#### 3.3.1. NaV1.2 and NaV1.1 Current Inhibition

**Figure 2:**
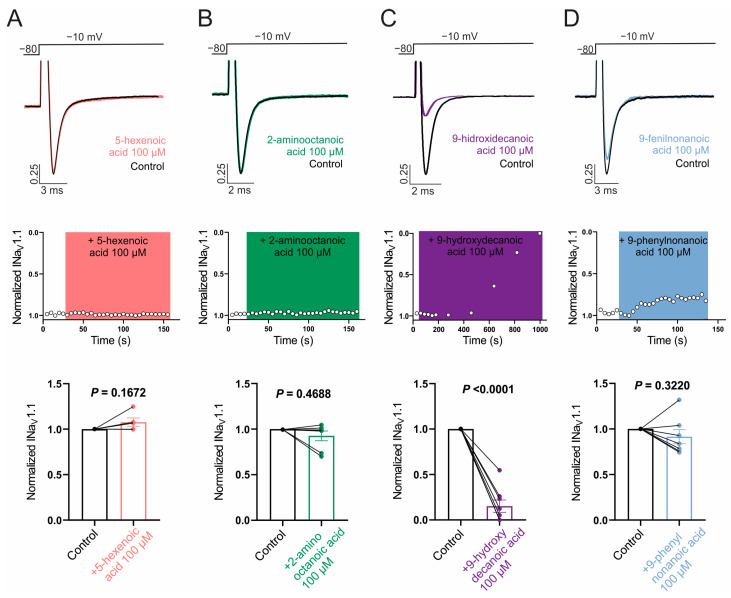
NaV1.1 current inhibition. Representative traces (**top**) and time courses (**middle**) of normalized NaV1.1 current (INaV1.1) from HEK293 cells with stable expression of NaV1.1 channels in control condition and 5-hexanoic acid (100 µM) application (n = 5, panel (**A**)); 2-aminooctanoic acid (100 µM) application (n = 7, panel (**B**)); 9-hydroxydecanoic acid (100 µM) application (n = 8, panel (**C**)); or 9-phenylnonanoic acid (100 µM) application (n = 7, panel (**D**)). Bars (**bottom**) represent normalized INaV1.1 in control condition and with each compound. Statistical significance was evaluated by one-sample *t*-test against 1.

**Figure 3:**
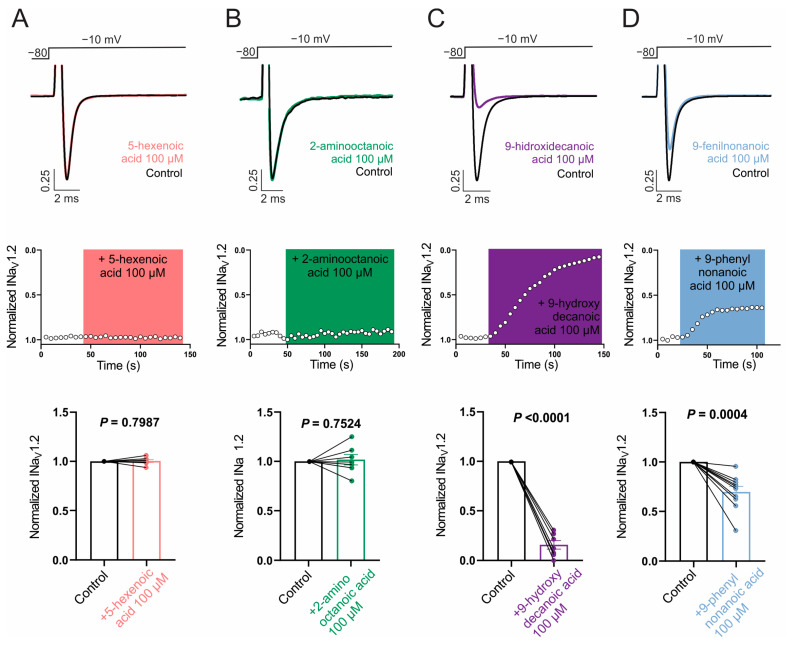
NaV1.2 current inhibition. Representative traces (**top**) and time courses (**middle**) of normalized NaV1.2 current (INaV1.2) from HEK293 cells with stable expression of NaV1.2 channels in control condition and 5-hexanoic acid (100 µM) application (n = 7, panel (**A**)); 2-aminooctanoic acid (100 µM) application (n = 7, panel (**B**)); 9-hydroxydecanoic acid (100 µM) application (n = 7, panel (**C**)); or 9-phenylnonanoicacid (100 µM) application (n = 10, panel (**D**)). Bars (**bottom**) represent normalized INaV1.2 in control condition and with each compound. Statistical significance was evaluated by one-sample *t*-test against 1.

Next, we evaluated the ability of the selected fatty acids to inhibit NaV1.1 and NaV1.2 channels by measuring its effect on Na+ currents evoked by a single test pulse in HEK cells stably expressing each isoform. Whereas NaV1.2 was our main target, it is of interest to evaluate the possible selectivity of NaV1.2/NaV1.1, since the NaV1.1 current is the major sodium current in specific inhibitory interneurons and its dysfunction is associated with certain types of epilepsies [64]. Figure 2A,B and Figure 3A,B show that neither 100 µM 5-hexenoic acid nor 2-aminooctanoic acid inhibited the Na+ currents of either channel isoform. In the case of 9-hydroxydecanoic acid, we observed a strong inhibition of 84.8 ± 6.7% and 84.2 ± 4.3% of the NaV1.1 and NaV1.2 currents, respectively (Figure 2C and Figure 3C). Despite the final inhibition being similar for the two isoforms (*p* = 0.9468, *t* test), we observed that the inhibition of NaV1.2 reached a stable value quickly, while the inhibition of NaV1.1 was gradual, taking more than 7 min to stabilize. Only 9-phenylnonanoic acid showed differences between the isoforms (Figure 2D and Figure 3D). This fatty acid exhibited a mild inhibition of 30.3 ± 5.6% on the NaV1.2 channel, with no significant effect on NaV1.1 (8.4 ± 7.8%, *p* = 0.322, one-sample *t*-test vs. 0%). Together, our results demonstrate that 9-hydroxydecanoic acid is a potent NaV channel inhibitor without isoform specificity. This last feature is present for the 9-phenylnonanoic acid that inhibits NaV1.2 without affecting NaV1.1 channels.

**Figure 4:**
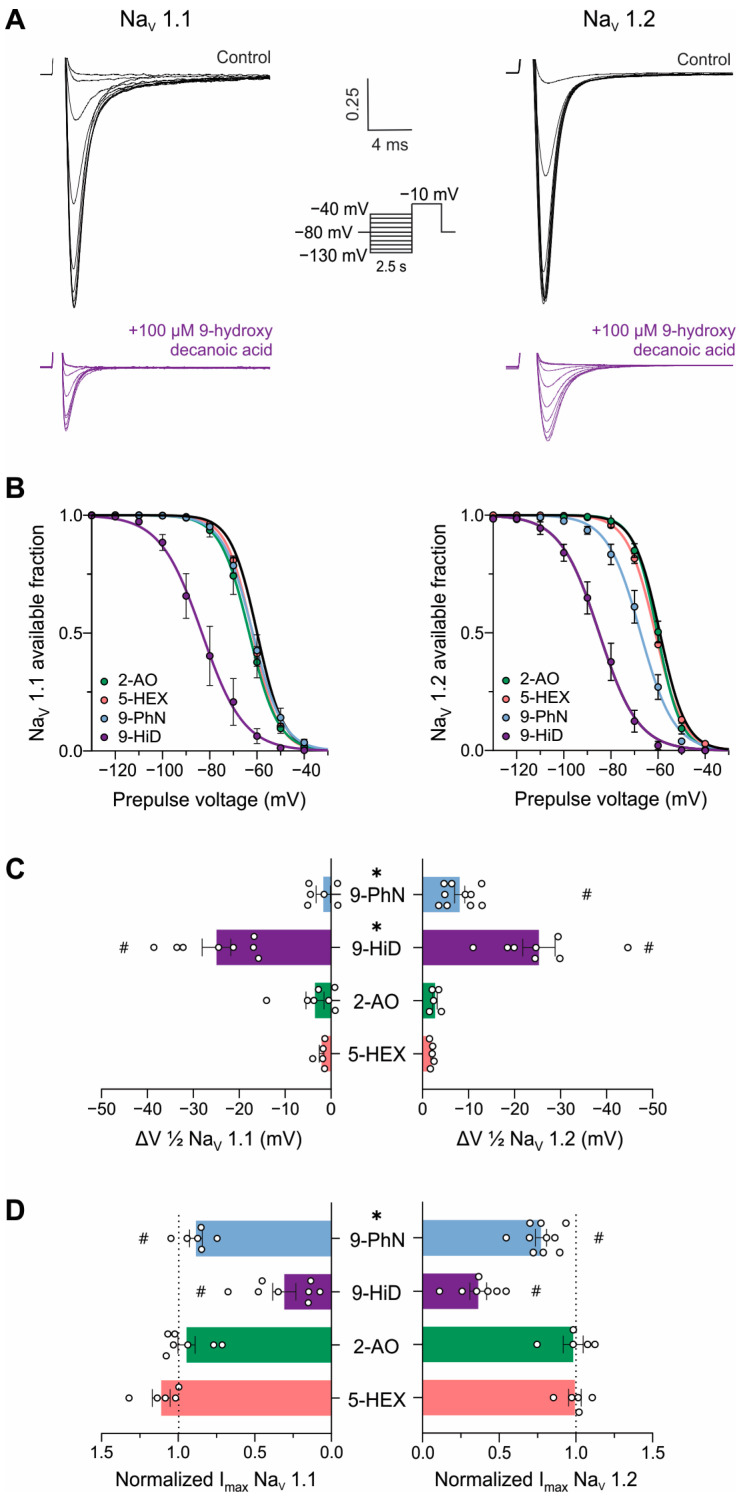
Mechanism of NaV channel inhibition by fatty acids. (**A**). Typical traces of human NaV1.1 (**left**) and NaV1.2 (**right**) currents evoked by the steady-state inactivation protocol under control conditions and after the application of 100 μM 9-hydroxydecanoic acid. (**B**). Mean h-curves obtained for control conditions and after stable effect of 100 µM 5-hexanoic acid (5-HEX, orange, NaV1.1: n = 5, NaV1.2: n = 5), 2-aminooctanoic acid (2-AO, green, NaV1.1: n = 7, NaV1.2: n = 5), 9-hydroxydecanoic acid (9-HiD, violet, NaV1.1: n = 8, NaV1.2: n = 7), and 9-phenylnonanoic acid (9-PhN, blue, NaV1.1: n = 6, NaV1.2: n = 10). (**C**). Mean change in half-maximal voltage inactivation (ΔV1/2) values obtained from the h-curves calculated for each fatty acid. # indicates statistically significant differences between ΔV1/2 values and 0 mV (one-sample *t*-test, *p* < 0.05); * indicates statistically significant differences between isoforms in the obtained ΔV1/2 values (unpaired *t*-test, *p* < 0.05). The slope parameter remained unchanged in all the treatments. (**D**). Mean voltage-independent inhibition, observed as a reduction in the Na+ current elicited after a pre-conditioning pulse of −130 mV (Imax) obtained after fatty acid treatment and normalized to control current value. # indicates statistically significant differences between Imax values and 1 (one-sample *t*-test, *p* < 0.05); * indicates statistically significant differences between isoforms in the obtained Imax values (unpaired *t*-test, *p* < 0.05).

To gain mechanistic insights into the observed NaV channel inhibition, we evaluated the effect of the fatty acids on the steady-state inactivation voltage protocol. Figure 4A shows typical recordings of Na+ currents of NaV1.1 and 1.2 isoforms obtained using this protocol before and after inhibition with 100 µM 9-hydroxydecanoic acid. This type of recording allows us to determine the fraction of channels available for opening at different pre-conditioning potentials (represented by the h curve). Drugs that bind to and stabilize the inactivated state of NaV channels shift the h curve to the left, as observed for 9-hydroxydecanoic acid on both tested isoforms and for 9-phenylnonanoic acid on NaV1.2 channels (Figure 4B,C). The application of 100 µM 9-hydroxydecanoic acid induced a statistically significant shift of 25.0 ± 3.1 and 27.3 ± 3.3 mV in the half-inactivation voltage parameter (V1/2) for the NaV1.1 and NaV1.2 isoforms, respectively. This shift was the greatest for all fatty acids tested in each isoform (NaV1.1: *p* = 0.0011, Kruskal–Wallis test; NaV1.2: *p* < 0.0001, ANOVA). In this state-dependent inhibition, the reduction in available fraction is greater when the cell membrane is more depolarized, leading to a voltage-dependent inhibition. This is a desired feature in anticonvulsant drugs, since it allows these drugs to preferentially block sodium channels in the overactive neurons involved in seizures, which tend to be more depolarized than healthy neurons. Moreover, in the typical recording shown in Figure 4A, it is possible to observe that 9-hydroxydecanoic acid reduces Na+ currents at pre-conditioning potentials where the available fraction of channels is maximal. This suggests an inhibitory effect independent of both the inactivated state and voltage. To quantify this inhibition, we measured the fatty acid’s effect on the current recorded during the test pulse following the −130 mV pre-conditioning pulse (Figure 4D). Consistent with the previous results, only 9-hydroxydecanoic acid and 9-phenylnonanoic acid significantly reduced the maximal current of h curves, with 9-hydroxydecanoic acid being the more potent inhibitor for both channel isoforms (NaV1.1 andNaV1.2: *p* < 0.0001, ANOVA). Notably, for this mechanism, the selectivity between the two channel isoforms is lost for 9-phenylnonanoic acid.

#### 3.3.2. CaV2.2 and CaV3.1 Current Inhibition

**Figure 5:**
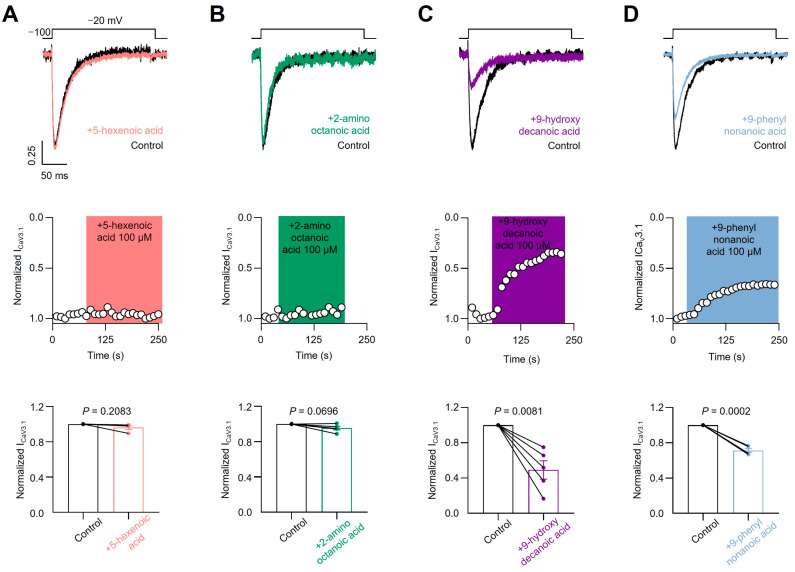
CaV3.1 current inhibition. Representative traces (**top**) and time courses (**middle**) of normalized CaV3.1 current (ICaV3.1) from HEK293T cells transfected with CaV3.1 in control condition and 5-hexanoic acid (100 µM) application (n = 4, panel (**A**)); 2-aminooctanoic acid (100 µM) application (n = 5, panel (**B**)); 9-hydroxydecanoic acid (100 µM) application (n = 5, panel (**C**)); or 9-phenylnonanoic acid (100 µM) application (n = 5, panel (**D**)). Bars (**bottom**) represent normalized ICaV3.1 in control condition and with each compound. Statistical significance was evaluated by one-sample *t*-test against 1.

**Figure 6:**
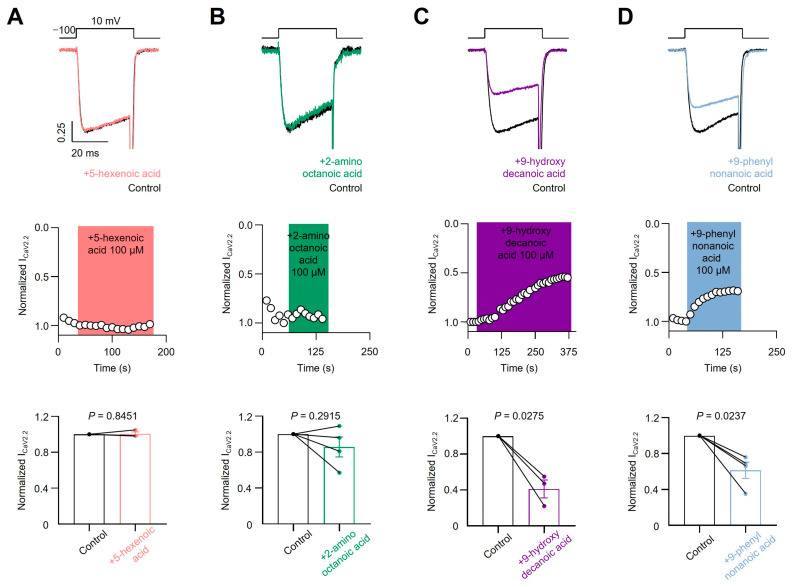
CaV2.2 current inhibition. Representative traces (**top**) and time courses (**middle**) of normalized CaV2.2 current (ICaV2.2) from HEK293T cells transfected with CaV2.2 in control condition and 5-hexanoic acid (100 µM) application (n = 3, panel (**A**)); 2-aminooctanoic acid (100 µM) application (n = 4, panel (**B**)); 9-hydroxydecanoic acid (100 µM) application (n = 3, panel (**C**)); or 9-phenylnonanoic acid (100 µM) application (n = 4, panel (**D**)). Bars (**bottom**) represent normalized ICaV2.2 in control condition and with each compound. Statistical significance was evaluated by one-sample *t*-test against 1.

To test the sensitivity of voltage-gated calcium channels to the four previously described fatty acids, we transfected HEK293T cells with the CaV3.1 subtype and acutely applied each compound. As shown in Figure 5A,B, we failed to observe a blocking effect of 5-hexenoic acid (100 µM) or 2-aminooctanoic acid (100 µM) on CaV3.1 macroscopic currents evoked at −20 mV from a holding potential of −100 mV. Next, we tested 9-hydroxydecanoic and 9-phenylnonanoic acids and observed significant CaV3.1 macroscopic current-blocking (Figure 5C,D). The estimated percentage of blocking for 9-hydroxydecanoic acid and 9-phenylnonanoic acid was 51.89 ± 10.40 and 28.82 ± 2.10, respectively. Then, we performed analogous experiments by transfecting HEK293T cells with the CaV2.2 subtype along with their auxiliary subunits CaVβ3 and CaVα2δ1 and measured the CaV2.2 macroscopic current. Similar to our observations with the CaV3.1 subtype, we failed to observe a blocking effect of 5-hexenoic acid or 2-aminooctanoic acid on CaV2.2 functionality at a concentration of 100 µM (Figure 6A,B). However, we observed that 9-hydroxydecanoic acid and 9-phenylnonanoic acid induced a significant blocking effect on CaV2.2 macroscopic currents recorded at +10 mV from a holding potential of −100 mV (Figure 6C,D). The percentage of blocking for 9-hydroxydecanoic acid and 9-phenylnonanoic acid was 58.72 ± 9.94 and 46.87 ± 8.04, respectively. Thus, we concluded that both 9-hydroxydecanoic acid and 9-phenylnonanoic acid acutely block the CaV3.1 and CaV2.2 macroscopic currents. Both targets have also been associated with epilepsies and seizures [65]. In particular, the gain of function variants of CaV3.1 have been implicated in childhood absence epilepsy.

### 3.4. Molecular Dynamics Simulations

**Figure 7:**
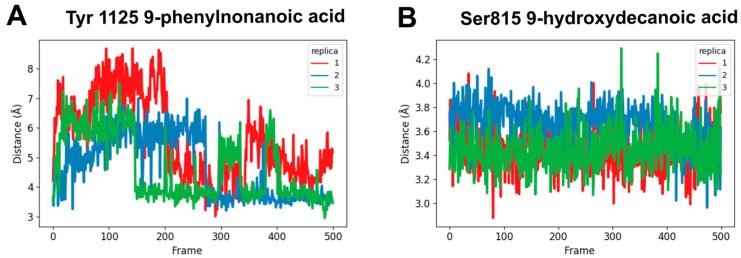
Molecular dynamics simulations. The distance between the carbonyl carbon of carboxylic acid and the oxygen of the protein side chain was monitored along 25 ns of simulation (n = 3). (**A**) 9-phenylnonanoic acid interaction with Tyr1125; (**B**) 9-hydroxydecanoic acid interaction with Ser815.

In order to better understand the binding interactions between the in vitro active compounds (9-phenylnonanoic acid and 9-hydroxydecanoic acid) and hNaV1.2, molecular dynamics simulations were performed. As the starting structures, we used the top scoring docking poses for the complexes. The simulations showed a high conformational flexibility for both candidates within the binding site, which is expected considering the relatively small size of the ligands and the presence of hydrocarbon chains responsible for nonspecific hydrophobic interactions towards the lipophilic environment of the NaV1.2 pore. The root-mean-square deviations (RMSD) along the simulations are shown in Figure S4. Despite their mobility, the candidates remained in the Ab site during the entire simulation. We also were able to identify a stable hydrogen bond interaction between the carboxylic function of 9-phenylnonanoic acid and Tyr1125 as well as a hydrogen bond interaction between the carboxylic group of 9-hydroxydecanoic acid and Ser815 (Figure 7).

## 4. Discussion

The neuroprotective role that fatty acids can play in relation to different insults to the brain, such as seizures or ischemia, has long been studied [66,67,68,69], which is in good agreement with the observation that under stress conditions, the amount of endogenous free fatty acids in the brain can increase dramatically [17]. These neuroprotective effects can be at least partially related to the well documented effect that fatty acids exert, directly or indirectly, on the activity of a variety of ion channels; these are effects that have been extensively investigated in cardiac ion channels [17,70]. For NaV and CaV channels, fatty acids tend to elicit a left-shift in the steady-state inactivation curve and, as a consequence, NaV and CaV channels are generally inhibited. The direct effects of fatty acids on ion channels would involve their interaction with the channel protein(s) or the surrounding membrane, whereas indirect effects would involve the prior biotransformation of the fatty acids to biologically active metabolites. Some reports with fatty acid analogs or inhibitors of biotransformation enzymes suggest that at least some fatty acids must be in their intact form to exert their effects on ion channels [70]; in other cases, the modulation of ion channels is exerted by fatty acid metabolites [71,72]. A major question is whether the reported effects of fatty acids on voltage-gated ion channels depend on the specific interaction of the fatty acid molecule with the channel proteins or if they are mediated via nonspecific membrane effects. In line with the fundamental assumption of the present study, which has undertaken the search of novel bioactive fatty acids and related molecules using molecular docking methods, several observations support a direct effect on the channel rather than a nonspecific membrane effect. First, the concentrations needed for the fatty acid effects are relatively low (in the low μM range) [73]. Second, no correlation has been found between the fatty acid’s propensity to fluidize the membrane and the effects on the channel conductance [74]. Third, soaking out cholesterol from the membranes affects ion channel function but does not modify the acute fatty acid effects [75]. Further, the onset and washout of the effect of fatty acids on ion channels is generally very rapid, also suggesting a direct channel effect [76].

Our results suggest that the structure-based in silico screening that we performed achieved a high predictive capacity, providing support to the underlying hypothesis of direct interaction of the fatty acids with the channel. Two of the four in silico hits submitted to in vitro assays corroborated their blocking capacity on the NaV1.2 sodium current. Although the number of hits evaluated is small, it can be highlighted that the rate of false positives (2/4, i.e., 50%) is relatively small, considering the class imbalance expected in any non-focused library subjected to screening. In general, the proportion of active compounds against the drug target of interest is expected to be small, while the anticipated proportion of inactive compounds is very high. This means that, even for models with a high predictive capacity and a low false positive rate, due to class imbalance, the number of false positives in the final list of in silico hits tends to be relatively high. We have included the dataset used in the validation of the model in the supplementary materials, which are available to the community.

It is noteworthy that the confirmed in silico hits are derived from saturated fatty acids, which provides new evidence that the structural requirements to modulate voltage-gated ion channels depend on the type of channel. In the case of voltage-gated potassium channels, for example, the structural features necessary to possess the modulating activity are associated with the length of the chain, the polyunsaturation of the double bonds in the acyl tail (with a preference for cis geometry), and the charge of the group head (which tends to disappear when the methyl- or ethyl-ester derivatives are obtained, which has been referred as the “lipoelectric mechanism”) [70,77]. In the case of sodium channels, initial studies by Xiao et al. using the whole-cell patch–clamp technique in HEK293t cells transfected with the α-subunit of the human cardiac Na+ channel (similarly to the in vitro system used in the current study) showed that both polyunsaturated and saturated fatty acids exerted inhibitory effects on the Na+ current [78]. Subsequently, though, the same group showed that the preference for polyunsaturated fatty acids reappeared if the α-subunit was co-expressed with the β-subunits in HEK293t cells, suggesting that the β-subunit may play a role in regulating lipid specificity in Nav channels [79]. This is consistent with the fact that Na+ currents are not affected by saturated or monounsaturated fatty acids in rat ventricular myocytes [80]. In the present study, two saturated fatty acid derivatives, 9-phenylnonanoic acid and 9-hydroxydecanoic acid, have shown inhibitory activity on sodium currents both in HEK293t cells transfected with the α-subunit, and also in vivo, in an acute model of seizure. The latter suggests that either the inhibitory effect of these hits on Na+ currents persist in presence of the β-subunits or that additional mechanisms of action are involved in the observed phenotypic antiseizure activity. Interestingly, the β-subunits in the brain differ in their expression and posttranslational modifications within different stages of development and in different tissues and cell types, which may confer pharmacological selectivity across different brain regions [81]. Thus, future efforts should focus on studying whether the effect of our in silico hits is preserved or not in the presence of different β-subunits.

According to our results, one of the confirmed hits (9-phenylnonanoic acid) showed NaV1.2/NaV1.1 selectivity, while the other confirmed hit (9-hydroxydecanoic acid) presents kinetic selectivity with a preference for NaV1.2 (whose blockade occurs much more quickly). These results are of interest considering the role of the NaV1.1 subtype in inhibitory interneurons [82].

In any case, the two corroborated hits also exhibited activity against CaV3.2 and CaV2.1, two calcium channels whose genetic variants have been associated with different types of epilepsy. This is not especially surprising, particularly considering the structural similarity between NaV1.2 and CaV3.2 and previous reports that various sodium current-blocking antiseizure drugs, such as phenytoin, ethosuximide, or zonisamide, also block T-type calcium channels [83,84].

Importantly, five sites of action of fatty acids have been described in relation to ion channels [70]: two of them are located in the ion-conducting pore, one at the intracellular entrance and the other at the extracellular entrance; the third is located at the voltage sensor domain-to-pore domain linker, close to the intracellular gate; and the last two are located at the interface between the extracellular part of the ion channel and the outer leaflet of the lipid bilayer. Since our structure-based virtual screening focused on the pore region, they should be complemented with studies on the other possible binding sites in the future, to further characterize the activity of our confirmed hits at the molecular level.

It is also interesting to note that although only two of the four compounds tested demonstrated the blockage of the sodium and calcium currents, the two inactive compounds in that assay also showed anticonvulsant activity in the MES assay; so, it remains to be determined what pharmacological target would be acting to produce its phenotypic effect. This result illustrates the complementary nature of screening focused on the pharmacological target and phenotypic screening. Since the second represents a target-agnostic strategy to identify new chemical entities with pharmacological potential, it allows the identification of compounds with unsuspected and, occasionally, novel pharmacological targets.

The activity that we report for these fatty acids identifies them as possible starting points for developing new drugs, and also arouses interest considering their potential inclusion in ketogenic foods with increased efficacy against epilepsies.

Finally, we would like to point out that the reported activity enables the perspective of evaluating our active hits against other pharmacological targets associated with epilepsy (in particular, the study of their effects on the GABAergic pathway is especially interesting considering that other fatty acids and derivatives have effects on that pathway) in other animal models of seizure and in animal models of epilepsy.

## Figures and Tables

**Table 1:** Anticonvulsant profiles on MES test. Data shown as the number of protected animals (animals that did not show a tonic extension of the hind limbs after electrical proconvulsant stimulus) over tested animals for each dose and time of administration. “Time” indicates how long before the application of the proconvulsant stimulus the tested drug was administered. For each time and dose two blocks were performed.

Compound	Dose	Time	
		0.5 h	4 h
5-Hexenoic acid	30 mg/kg	2/5	3/5
	100 mg/kg	0/5	3/5
2-Aminooctanoic acid	30 mg/kg	4/5	4/5
	100 mg/kg	4/5	5/5
9-Hydroxydecanoic acid	30 mg/kg	3/5	4/5
	100 mg/kg	4/5	3/5
9-Phenylnonanoic acid	30 mg/kg	0/5	2/5
	100 mg/kg	2/5	4/5

## Data Availability

The original contributions presented in the study are included in the article/Supplementary Materials; further inquiries can be directed to the corresponding author/s.

## Supplementary Materials

The following supporting information can be downloaded at: https://www.mdpi.com/article/10.3390/pharmaceutics16080996/s1, Figure S1: area under the receiver operating characteristic curve (AUC-ROC) for the docking protocol validation. Figure S2: 2D representations of the top 10 docked complexes based on the scoring function. Figure S3: 3D representations of the top 10 docked complexes based on the scoring function. Figure S4: Molecular dynamics simulations for 9-phenylnonanoic acid and 9-hydroxydecanoic acid. Table S1: Docking protocol validation dataset. Table S2: Specific pathogen free in tested mice. Table S3: Docking scores.
