# Supplementary figures and images for: In Silico Screening Identification of Fatty Acids and Fatty Acid Derivatives with Antiseizure Activity: In Vitro and In Vivo Validation
Source: Pharmaceutics. 2024 Jul 27;16(8):996. doi: 10.3390/pharmaceutics16080996 (PMC11357650; doi:10.3390/pharmaceutics16080996)

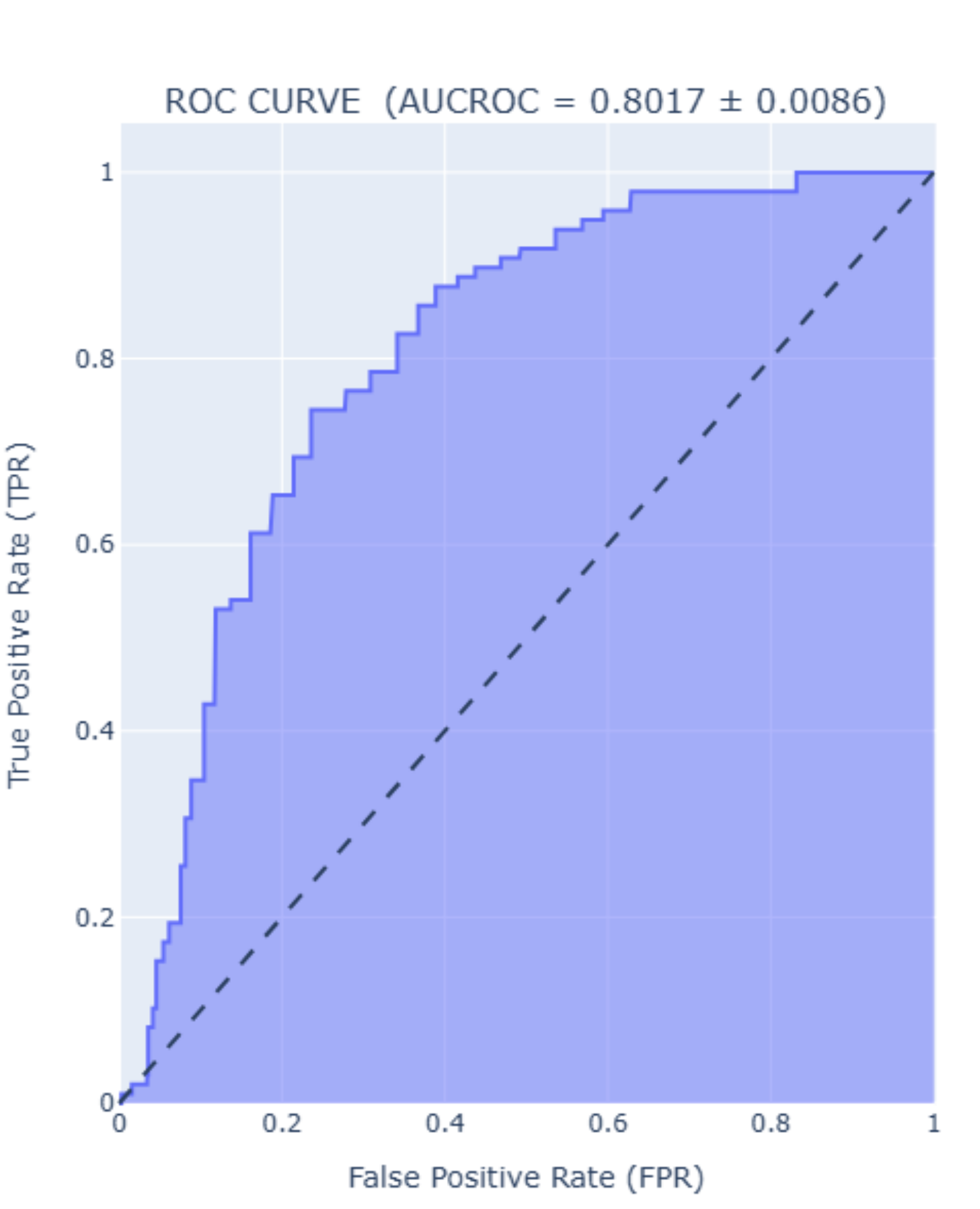

Supplement: Supplementary file 1 [file pharmaceutics-16-00996-s001.zip › Figure S1 (1).png]

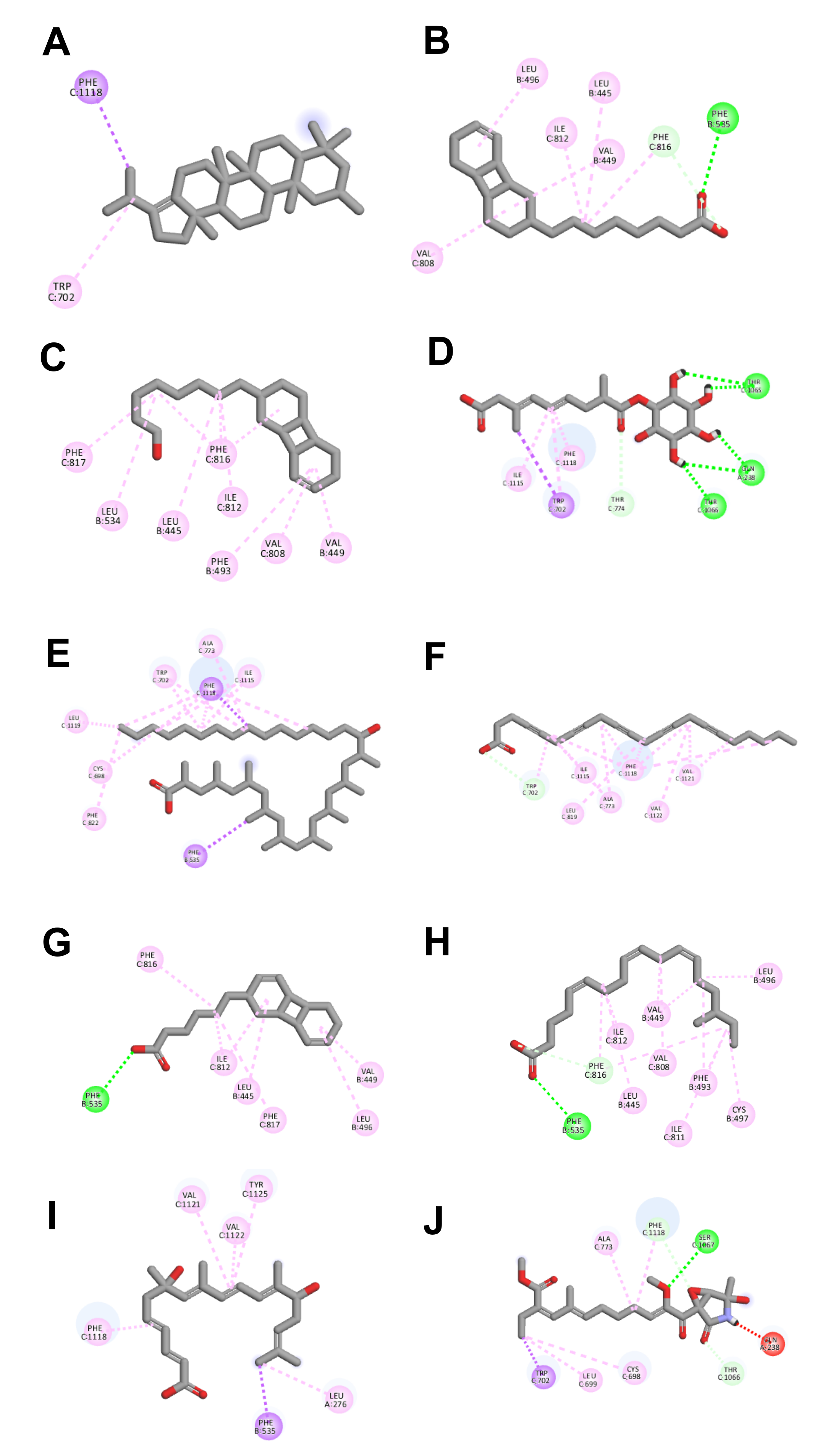

Supplement: Supplementary file 1 [file pharmaceutics-16-00996-s001.zip › Figure S2_Vrev.png]

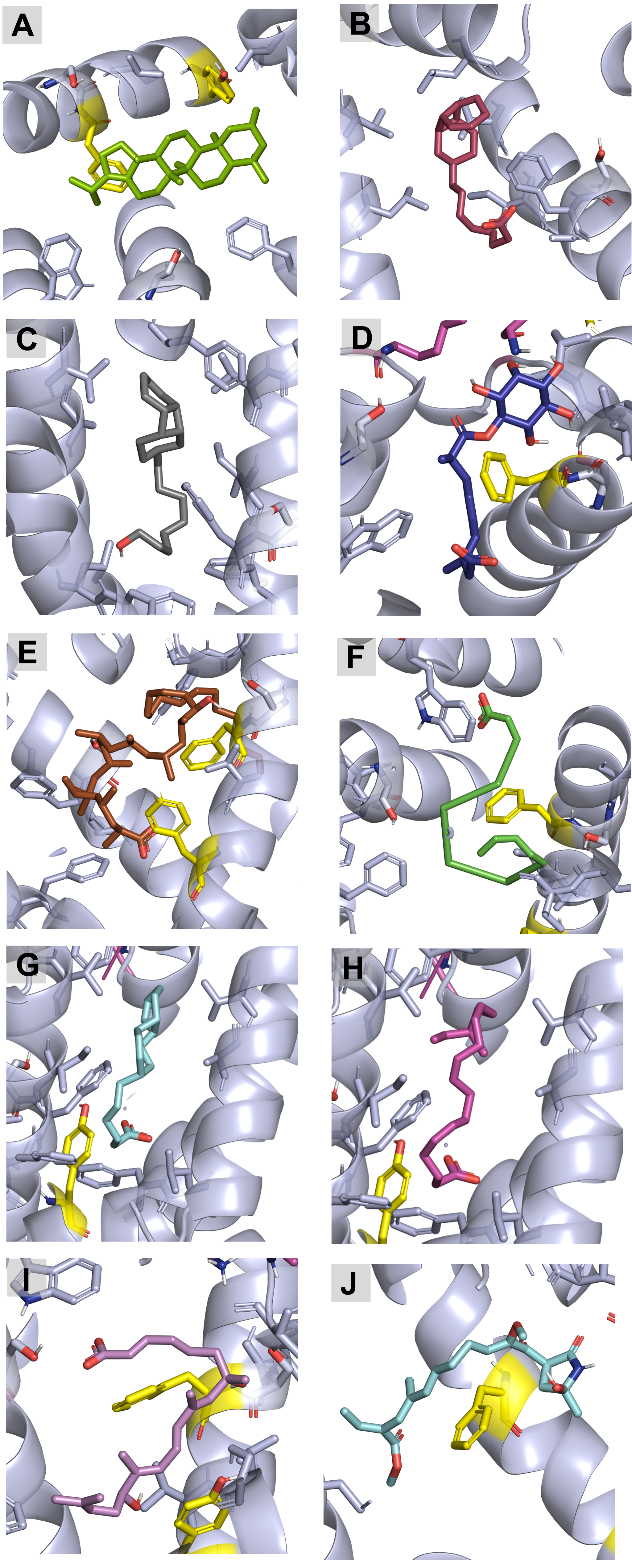

Supplement: Supplementary file 1 [file pharmaceutics-16-00996-s001.zip › Figure S3_Vrev.png]

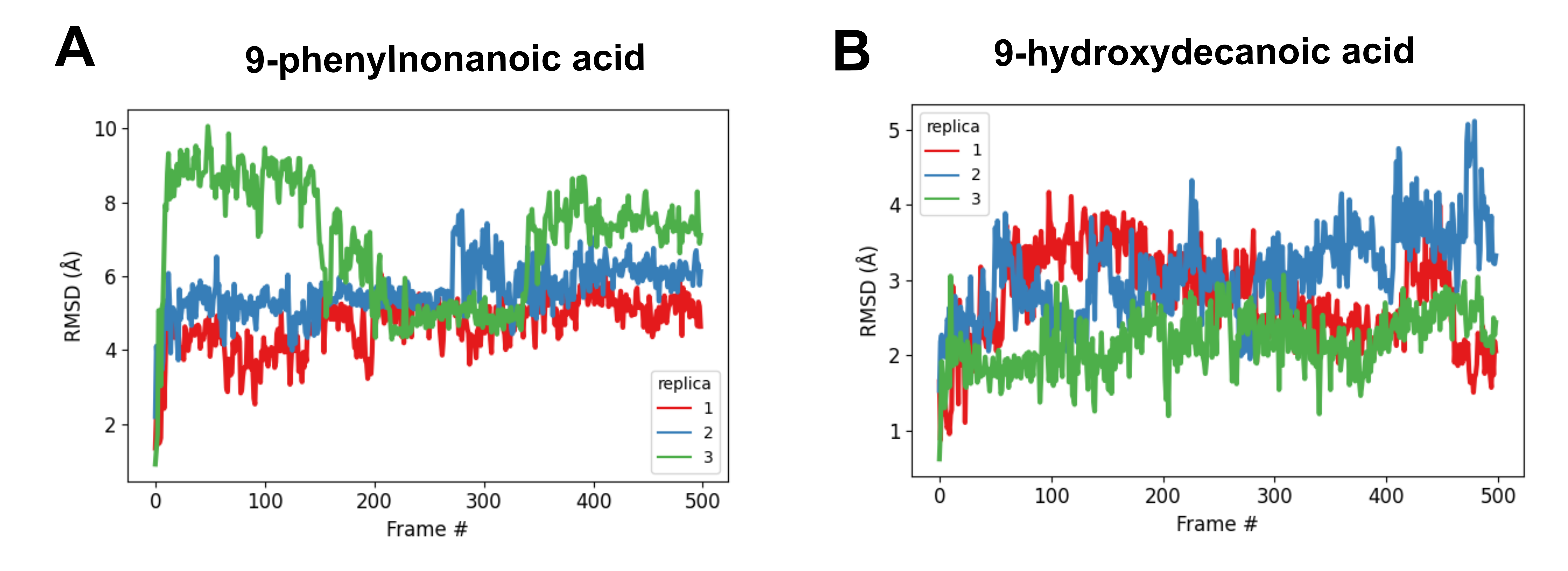

Supplement: Supplementary file 1 [file pharmaceutics-16-00996-s001.zip › Figure S4_Vrev.png]
